# Leachability and Anti-Mold Efficiency of Nanosilver on Poplar Wood Surface

**DOI:** 10.3390/polym14050884

**Published:** 2022-02-23

**Authors:** Xiaohan Dai, Yanran Qi, Hongxue Luo, Zaixin He, Lianxiang Wei, Xiaoying Dong, Xingxia Ma, De-Quan Yang, Yongfeng Li

**Affiliations:** 1 Key Laboratory of State Forestry Administration for Silviculture of the Lower Yellow River, College of Forestry, Shandong Agricultural University, Taian 271018, China; dxiaohan0315@163.com (X.D.); qyran1994@163.com (Y.Q.); luohongxue1997@163.com (H.L.); hzaixin@126.com (Z.H.); 15662006529@163.com (L.W.); 2 Research Institute of Wood Industry, Chinese Academy of Forestry, Beijing 100091, China; 3 Solmont Technology Wuxi Co., Ltd., 228 Linghu Blvd., Wuxi 214135, China; derry.yang@solmontech.com

**Keywords:** silver nanoparticle, wood surface, mold, protective efficiency, leachability

## Abstract

Water-based antimicrobial agents, used in environmentally friendly applications, are widely used in wood protection industries. Furthermore, nanomaterials as antimicrobial agents, because of their biocidal component, huge specific surface area, and unique nanoscale effect, have attracted attention in the field of biodurability. We employed aqueous dispersed nano-silver with a diameter of 10 nm~20 nm to treat poplar wood and evaluated its leaching resistance and anti-mold effect on the wood surface. The results revealed that the higher the retention of the nano-silver, the stronger the protection efficiency of the wood surface against three molds (*Aspergillus niger V.* Tiegh, *Penicillium citrinum* Thom, and *Trichoderma viride Pers.* ex Fr); and the leachability of the nano-silver presented a slowly growing trend with the increase in the retention. When the wood surface attained a silver retention of 0.324 g·m^−2^, its anti-mold efficiency against *Aspergillus niger V.* Tiegh, *Penicillium citrinum* Thom, and *Trichoderma viride Pers.* ex Fr reached 80, 75, and 80%, respectively, which achieved or even exceeded the required standard value of effective mold inhibition (75%). Notably, the nano-silver leaching rate at this retention attained merely 4.75 %. The nanoparticle, well distributed on a wood surface, may promote sufficient contact with fungi as well as strong interaction with wood cell wall components, which probably contributed to the effective anti-mold efficiency and the leaching resistance. This study provided positive evidence for the anti-mold effect of nano-silver on wood surface.

## 1. Introduction

Wood, as a natural and renewable material widely used in construction and home decoration, possesses the advantages of beautiful texture, gentle touch, high strength-to-weight ratio, and biodegradability; thus, is positioned as an ecomaterial suitable for sustainable development in the fields of social economy as well as the realization of “carbon neutralization” goals [1,2]. However, when wood is used in humid environments, mold infection can easily to occur on its surface, which adversely affects the aesthetic quality of the wood, and can even release spores into surroundings, potentially polluting the environment and endangering human health [3,4,5,6]. Therefore, anti-mold protective treatment on wooden surfaces is an effective way to improve the decorative quality of the ecomaterial and the living environment where the materials are applied, which is important in the promotion of the wide and efficient utilization of wood ecomaterials [7].

Generally, water-borne anti-mold agents, featured in green processes, could effectively resist mold infestation on wood surfaces, such as didecyl dimethyl ammonium chloride, borates, and quaternary ammonium salts [8,9,10,11]. However, they are susceptible to migration and leaching when applied in humid environments, resulting in reduced anti-mildew efficiency and potential environmental pollution [12]. Therefore, developing environment-friendly anti-mold agents with combined excellent antifungal efficiency and leaching resistance is still a challenge in the field of wood mildew-proofing. Fortunately, antimicrobial nanoparticles, such as nano-silver (Ag), -copper (Cu), -zinc oxide (ZnO), and -titanium dioxide (TiO_2_), merge the advantages of their antifungal effect due to the high specific surface area, many active sites, small size effect, biocidal properties, and thermal stability, as higher leaching resistance than water-soluble ions, which make them regarded as an emerging generation of anti-mold agents [13,14,15].

Nanometal particles, such as nano-silver and nano-copper, inhibit microbial growth mainly by contacting microbial cells and destroying their intracellular structure. Research shows that wood and bamboo impregnated with nano-silver and nano-copper effectively inhibited mold growth [16,17,18,19,20]. Nano-zinc oxide and nano-titanium dioxide, possessing photocatalytic effects under conditions of ultraviolet light irradiation and oxygen, could produce reactive oxygen species to kill microorganisms. A study reports that aqueous dispersal of nano-zinc oxide (100 nm) with 0.1 wt.% concentration effectively inhibited the breeding of *Aspergillus niger*, *Trichoderma harzianum,* and *Penicillium pinophilus* on the surface of scotch pine wood within 4 weeks and presented good leaching resistance [13]; and aqueous dispersal of nano-titanium dioxide coated on the surfaces of softwood and hardwood effectively inhibited the growth of *Aspergillus niger* [14]. The combined utilization of these nanomaterials may also exert a synergistic function, which could further improve the mildew prevention efficiency [21]. For example, combined nano-Ag, Cu, and TiO_2_ (40–50 nm) particles coated on softwood and hardwood surfaces significantly inhibited the reproduction of *Aspergillus Niger* [14]; combined treatment of TiO_2_ nanoparticles and petal-shaped nano-ZnO on a bamboo surface endowed the material with an anti-mold efficiency of grade zero against a mixture of *Aspergillus niger*, *Trichoderma viride,* and *Penicillium citrinum* [22]; nano-Ag doped into TiO_2_ film, which was in situ produced on a bamboo surface, imparted excellent antifungal activity to the material, which presented no obvious hyphae of *Trichoderma viride* and *Penicillium citrinum* [23]. In addition, some studies showed that the antifungal nanomaterials exhibited good leaching resistance on wood surface, which contributed to the antibacterial efficiency [24]. Each nano-copper or nano-zinc treated southern pine wood showed both leaching resistance and mold inhibition [25]. Pine sapwood treated by nano-silver with a concentration of 3 g·L^−1^ exhibited a lower leaching rate of biocide of about 15% [26]. Briefly, these studies indicate that the above antifungal nano-metals appear to inhibit mold and also leaching resistance.

Although previous reports revealed that the particle size, biocidal components’ retention, and the actuation duration of the antifungal nanomaterials had significant effects on mold growth, there was a lack of adequate realization for the relationship between mold inhibition and the nanomaterials’ function, such as linkage among protective efficiency, retention, and leaching rate [16,27]. Especially, nano-silver as an anti-mold inhibitor has been widely studied. However, the relationship among inhibition, retention, and particle size of the nano-Ag against wood molds has not been systematically investigated. Therefore, such a study is novel and meaningful. This study employed aqueous dispersed nano-silver (Nano-Ag) with diameter of 10 nm~20 nm to treat a poplar wood surface for mold inhibition. The variations of the leaching resistance and the anti-mold effect of the nano-Ag on wood surface against the silver retention were primarily explored, and the appropriate retention of the nano-Ag to effectively inhibit the mold growth on wood surface was further determined, which provides a scientific basis for the application of nano silver in anti-mold treatment of wood surfaces.

## 2. Materials and Methods

### 2.1. Materials

Anti-mold nano-Ag particles had sphere-like morphology with diameters ranging from 10 nm to 20 nm, and were stably suspended in aqueous solution with PVP (polyvinyl pyrrolidone)-PVA (polyvinyl alcohol) mixing, which was provided by Solmont Technology Inc. (Wuxi, China). Poplar wood (0.386 g·cm^−3^), purchased from the local timber market of Tai’an city, were cut into specimens with dimensions of 50 mm × 20 mm × 3 mm (L × T × R) and pad-wood samples with dimensions of 60 mm × 6 mm × 3 mm (L × T × R), which had no bugs, knots, or other defects. Three microorganism strains were purchased from the China Forestry Collection Center (CFCC) (Beijing, China) for *Aspergillus niger V.* Tiegh. (CFCC 82449) and *Pencillium citrinum* Thom (CFCC 89234), and Agricultural Culture Collection of China (ACCC) (Beijing, China) for *Trichoderma viride Pers.* ex Fr. (ACCC 30595).

### 2.2. Methods

#### 2.2.1. Mold Resistance of Nano-Ag in Culture Medium and Its Concentration Screening

First, the 3 fungal strains were cultured on a PDA substrate within Petri dishes (standard 90 mm) for 7 days under conditions of 28 °C and 85% relative humidity. Then, the nano-Ag dispersed solution was added into pure PDA substrate to obtain the nano-Ag loaded culture medium with concentrations of 2 ppm (0.002 kg·m^−3^), 20 ppm (0.02 kg·m^−3^), 200 ppm (0.2 kg·m^−3^), 500 ppm (0.5 kg·m^−3^), and 700 ppm (0.7 kg·m^−3^); while the control group (CK) was pure PDA substrate without nano-Ag loading. After 7 days cultivation, the fragments with mycelia were cut from the edge of the fungal colonies and inoculated in the center of nano-Ag loaded substrates. Finally, all the experimental specimens were cultured at 28 °C and 85% relative humidity for 28 days. During the incubation, the growth diameters of the fungal colonies were measured every week and the inhibition rate was calculated by Equation (1). The datum was the mean value of 5 replicates.

(1) $$Inhibition\;rate\;\left(\%\right)=\left(\frac{D_{\mathrm{control}}-D_{\mathrm{experiment}}}{D_{\mathrm{control}}}\right)\times 100$$

where *D*_control_ is the average diameter of mycelium grown on the control plate, mm; *D*_experiment_ is the average diameter of mycelium grown on the nano-Ag plate, mm.

#### 2.2.2. Treatment of Poplar Wood by Nano-Silver and the Leaching Evaluation

Briefly, the nano-Ag dispersed solution was first diluted into concentrations of 500 ppm, 700 ppm, and 1000 ppm with deionized (DI) water. Next, the wood specimens were dried to constant weights and marked as m_1_, and then immersed into the nano-Ag dispersed solution with different concentrations and distilled water (control) for 3 min, respectively; and finally left to stand for overnight, then weighed again (*m*_2_). Each experiment was conducted in 6 parallels. The nano-Ag retention of wood surface was calculated by Equation (2).

(2) $$R=\frac{\left(m_{2}-m_{1}\right)\times c}{2\left(L\times H+L\times W+H\times W\right)}\times 10^{6}$$

where *R* is the nano-Ag retention of wood surface, g·m^−2^; *m*_1_ is the sample weight before immersion, g; *m*_2_ is the sample weight after immersion, g; *c* is the concentration of nano-Ag dispersed solution, ppm; *L* is the sample length, mm; *W* is the sample width, mm; and *H* is the sample height, mm.

According to the Chinese Standard, GB/T 29905-2013 [28], the leaching rate of different nano-Ag loaded wood samples were evaluated. First, each group of the treated wood with six specimens was immersed in 180 mL DI water within individual 500 mL beaker. Then, the samples were subjected to mild agitation for 14 days, and the distilled water was renewed after 6 h, 24 h, 48 h, and at 4-, 6-, 8-, 10-, 12-, and 14-day intervals. Meanwhile, all the leachates were collected and blended. Finally, the sliver ion content was measured by the Inductively Coupled Plasma-Mass Spectrometry (ICP-MS). Briefly, the sample of leachate solution was first dissolved with nitric acid, then fixed to 100 mL, from which 10 mL was taken and diluted into 100 mL with deionized water, and finally the standard sample was configured to make the standard curve for testing. The leaching rate (*L*) was calculated by Equations (3) and (4), and the anti-leaching rate (*L_A_*) was calculated by Equation (5).

(3) $$m=c\times m_{G}\times 1000$$

(4) $$L\;\left(\%\right)=\frac{m_{A}}{m}\times 100$$

where *m* is the total mass of the silver elements in the wood samples, mg; *c* is the mass fraction of silver elements in the dispersed solution, %; *m_G_* is the mass of the nano-Ag dispersed solution absorbed by the wood samples, g; *m_A_* is the mass of silver element in the leachate, mg.

(5) $$L_{A}\;\left(\%\right)=\left(1-L\right)\times 100=\left(1-\frac{m_{A}}{m}\right)\times 100$$

#### 2.2.3. Anti-Mold Evaluation of Nano-Ag Treated Wood Materials

Anti-mold properties of the wood samples were evaluated according to the Chinese Standard GB/T 18261-2013 [29]. Briefly, the fungal strains were first cultured on PDA substrate within Petri dishes for 7 days at 28 °C and 85% relative humidity. Then, the nano-Ag dispersed solution was prepared into concentrations of 500, 700, and 1000 ppm, with DI water. The poplar wood specimens were treated by the nano-Ag dispersed solution according to the above steps (Section 2.2.2), and the nano-Ag retention of wood surface was calculated by Equation (2).

After the PDA substrate within Petri dishe was covered by the fungal strains, two pad-wood samples were placed on the PDA substrate. One was for a control sample and the other one was for the nano-Ag treated wood sample. After inoculation, the fungi were cultured at 28 °C and 85% relative humidity for 28 days. During the incubation, the infection area of the fungal growth was measured every 7 days, and the corresponding infection value of the mold was calculated according to the Table 1. Finally, the protection efficiency (*E*) of the nano-Ag against mold infection on wood samples was obtained from Equation (6).

(6) $$E\left(\%\right)=\left(1-\frac{D_{1}}{D_{0}}\right)\times 100$$

where *D*_1_ is the average infection value of the wood samples; *D*_0_ is the mean infection value of the control group.

#### 2.2.4. Characterization Methods

The morphologies of nano-Ag and wood samples before and after mold infection were characterized by scanning electron microscopy (SEM) (JSM-6610LV, JEOL, Tokyo, Japan), field-emission scanning electron microscopy (FESEM) (GeminiSEM 300, Carl Zeiss, Dresden, Germany), and transmission electron microscopy (TEM) (JEM-1400 plus, JEOL, Tokyo, Japan).

## 3. Results

### 3.1. Preliminary Screening of Nano-Ag Concentration in Culture Medium

The nano-Ag loaded culture medium samples with concentrations of 2 ppm, 20 ppm, 200 ppm, 500 ppm, and 700 ppm were labeled as Ag2, Ag20, Ag200, Ag500, and Ag700, respectively; while the control group (i.e., the pure PDA substrate) was labeled as CK. All these were explored to preliminarily screen the nano-Ag concentration range of effectively inhibiting the mold growth in the culture medium, which could provide scientific evidence for the anti-mold study of nano-Ag on wood surface.

**Figure 1:**
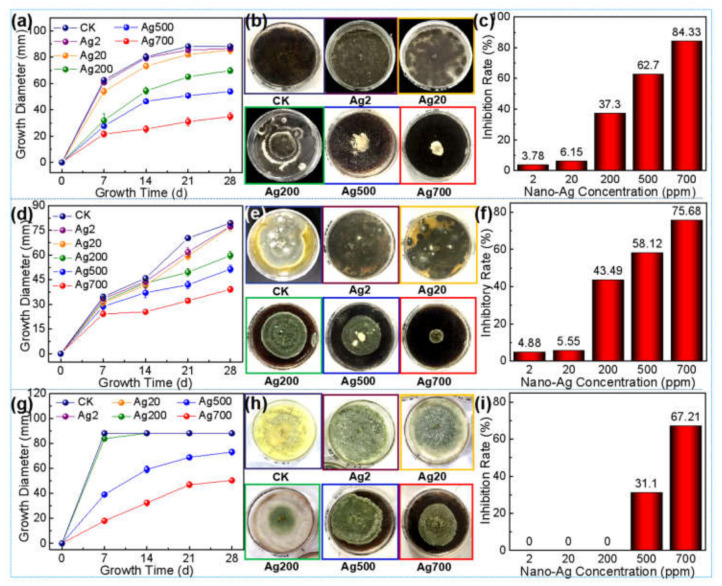
Growth inhibition of the three molds by nano-Ag and control group (CK) in the medium: (**a**) Growth of *Aspergillus niger* with time at different nano-Ag concentrations (CK, Ag2, Ag20, Ag200, Ag500, Ag700); (**b**) Digital photos of *Aspergillus niger* after growth for 28 days at different nano-Ag concentrations; (**c**) Growth inhibition rate of *Aspergillus niger* at different nano-Ag concentrations; (**d**) Growth of *Penicillium citrinum* with time at different nano-Ag concentrations; (**e**) Digital photos of *Penicillium citrinum* after 28 days of growth at different nano-Ag concentrations; (**f**) Growth inhibition rate of *Penicillium citrinum* at different nano-Ag concentrations; (**g**) Growth of *Trichoderma viride* with time at different nano-Ag concentrations; (**h**) Digital photos of *Trichoderma viride* after 28 days of growth at different nano-Ag concentrations; (**i**) Growth inhibition rate of *Trichoderma viride* at different nano-Ag concentrations.

Figure 1 shows that the growth diameters of all the three molds increased with time on each PDA substrate. For the *Aspergillus niger*, its growth diameter on the CK PDA substrate was still larger than that of each nano-Ag loaded substrate during the 28-day growth period; and its mycelia growth diameter decreased with the increase in the nano-Ag concentration; among them, the mycelia growth diameters of both the Ag2 and Ag20 groups were slightly lower than that of the CK group, indicating weak inhibition of lower-concentrated nano-Ag against the *Aspergillus niger* growth; when the nano-Ag concentration was ≥200 ppm, the growth diameter was significantly lower than that of the CK group, indicating that such concentrated nano-Ag had a certain inhibitory effect on the fungus growth. After the fungus grown on the medium for 28 days, the inhibition efficiency (i.e., the inhibition rate) of the Ag500 and Ag700 groups achieved 62.7% and 84.33%, respectively, which was higher than 50%, indicating to some extent that ≥500 ppm was an effective concentration range of inhibiting the fungus growth on PDA substrate (Figure 1a–c).

The growths of *Penicillium citrinum* and *Trichoderma viride* on the nano-Ag loaded mediums with different concentrations were similar to that of *Aspergillus niger*, both of which presented a gradually decreased growth diameter with the increase in the nano-Ag concentration (Figure 1d,e,g,h), i.e., their growths were increasingly inhibited. For the *Penicillium citrinum*, the inhibition rate of Ag500 and Ag700 group reached 58.12% and 75.68%, respectively, indicating that ≥500 ppm seemed to be an effective concentration range in inhibiting the fungus growth on the medium (Figure 1f); while for the *Trichoderma viride*, only the Ag700 group showed a higher inhibition efficiency of over 50%, which indicated an obvious anti-mold effect; i.e., ≥700 ppm was the relatively effective anti-fungus concentration range of nano-Ag against the *Trichoderma viride* growth on the medium (Figure 1i).

In conclusion, the nano-Ag with concentration ranges higher than 500 ppm, 500 ppm, and 700 ppm could effectively inhibit the growth of *Aspergillus niger*, *Penicillium citrinum,* and *Trichoderma viride*, respectively, on the PDA substrate, which suggests the concentration of nano-Ag should be designed as 500 ppm, 700 ppm, and 1000 ppm for the following experiments of all three molds’ inhibition on wood surface.

### 3.2. Evaluation of Leachability of Nano-Ag on Wood Surfaces

**Figure 2:**
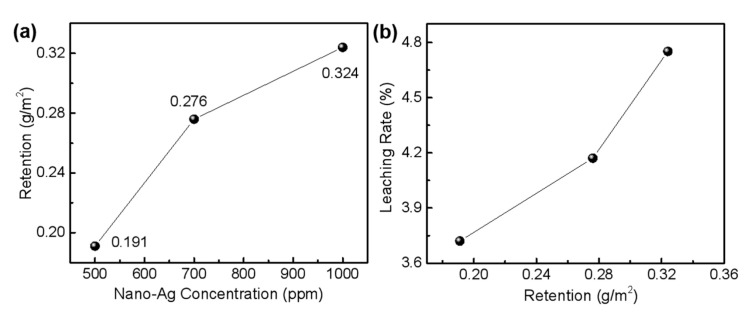
Relationships among retention and leaching rates of nano-Ag on wood surface and its concentration: (**a**) variation of nano-Ag retention with its concentration; (**b**) variation of nano-Ag leaching rate with its retention.

Figure 2 showed that under the employed experimental conditions, the nano-Ag retention on wood surface increased with its concentration (Figure 2a), and the leaching rate of the nano-Ag increased with its surface retention (Figure 2b), which was consistent with the results reported in literature [26,27]. When the nano-silver concentration was 500 ppm, its retention on wood surface was 0.191 g·m^−2^, and the leaching rate reached 3.72%, i.e., the anti-leaching rate was 96.28%. When the concentration of nano-silver was 1000 ppm, the wood retention attained 0.324 g·m^−2^, and the leaching rate reached 4.75%, i.e., the anti-leaching rate achieved 95.25%, which indicates that the nano-Ag has better leaching resistance on wood surface.

### 3.3. Evaluation of Anti-Mold Effect of Nano-Ag on Wood Surface

**Figure 3:**
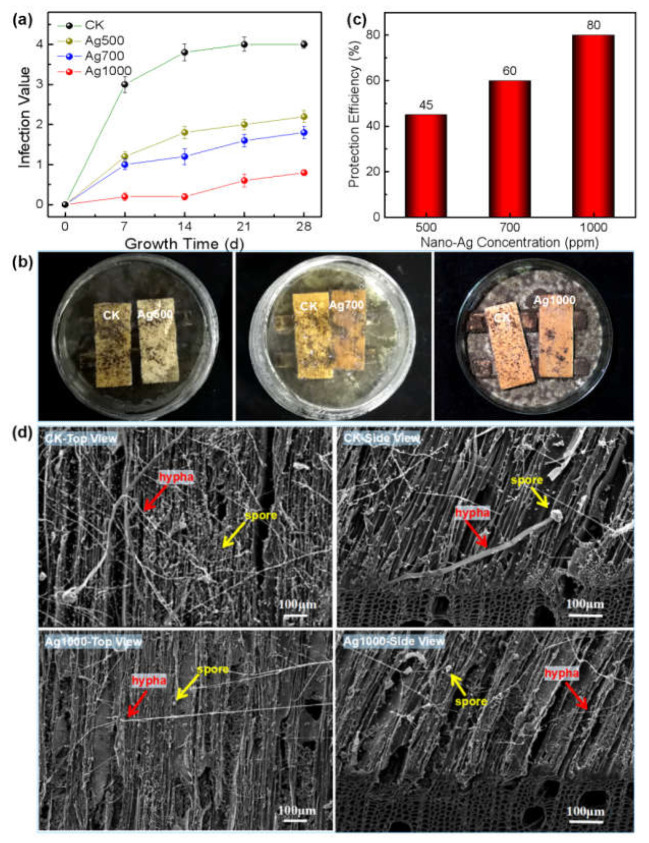
Growth inhibition of nano-Ag against *Aspergillus niger* on wood surface: (**a**) Variation of infection values of *Aspergillus niger* on wood surface with time under different nano-Ag concentrations (correspond to retentions, shown in Figure 2); (**b**) Digital photos of *Aspergillus niger* on wood surface after 28-day growth period under different nano-Ag concentrations (in the same Petri dish: left—CK; right—nano-Ag treated sample); (**c**) Protection efficiency of nano-Ag against *Aspergillus niger* on wood surface under different nano-Ag concentrations; (**d**) SEM morphologies of the surfaces of CK wood and the wood treated by nano-Ag with 1000 ppm concentration after the fungal infection.

The results presented in Figure 3 show that the infection values of *Aspergillus niger* increased with its infestation time on both CK wood and the nano-Ag loaded wood surfaces; and all the values of nano-Ag loaded wood were correspondingly lower than that of the CK wood, indicating improved fungi resistance due to the nano-Ag function (Figure 3a). Additionally, the infection value decreased sequentially with the increase in nano-Ag concentration (i.e., the nano-Ag retention). After the 28-day growth period, the fungus growth area on each wood surface, loaded with different concentrations of nano-Ag particles, was obviously lower than that of the CK wood (Figure 3b). When the nano-Ag concentration reached 1000 ppm (i.e., the nano-Ag retention reached 0.324 g·m^−2^, shown in Figure 2a), the protection efficiency of the nano-Ag loaded wood surface reached 80%, higher than the standard value of 75%. Thus, 1000 ppm was the toxic threshold concentration of nano-silver inhibiting *Aspergillus niger* growth on wood surface (GB/T 18261-2013 [29]) (Figure 3c). Under such conditions, there were only a small number of spores and hypha present on the nano-Ag loaded wood surface (Ag1000-top view of Figure 3d), and rare growth traces of the fungus left below the surface layer (Ag1000-side view of Figure 3d), indicating that nano-Ag effectively inhibited the mold from infesting the wood surface. In contrast, the CK wood surface was covered with numerous spores and slender hypha (CK-top view of Figure 3d), which corresponded to the macroscopic appearance of the CK wood surface presented in Figure 3b; and similarly, the mycelia were almost invisible below the surface layer of 100 μm (CK-side view of Figure 3d), suggesting that the fungus only infested a thin layer of the wood surface, which normally would not affect the structure and strength of wood [30]. The above results indicate that the nano-silver treatment with 1000 ppm concentration can effectively inhibit the fungus reproduction on wood surface.

**Figure 4:**
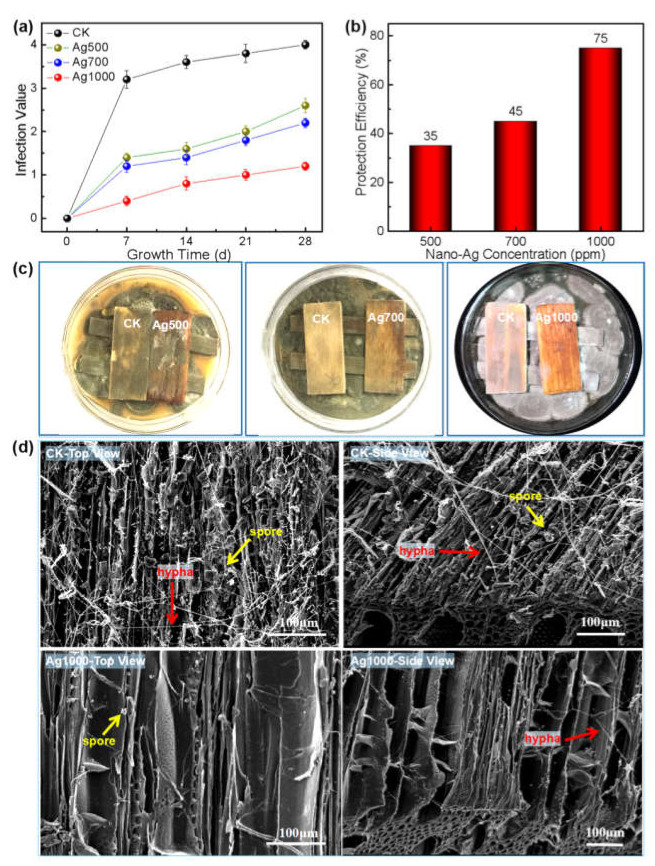
Growth inhibition of nano-silver against *Penicillium citrinum* on wood surface: (**a**) Variation of infection values of *Penicillium citrinum* on wood surface with time under different nano-silver concentrations (correspond to retentions, shown in Figure 2); (**b**) Protection efficiency of nano-silver against *Penicillium citrinum* on wood surface under different nano-silver concentrations; (**c**) Digital photos of *Penicillium citrinum* on wood surface after 28-day growth period under different nano-silver concentrations (in the same Petri dish: left—CK; right—nano-silver treated sample); (**d**) SEM morphologies of the surfaces of CK wood and the wood treated by nano-silver with 1000 ppm concentration after *Penicillium citrinum* infection.

**Figure 5:**
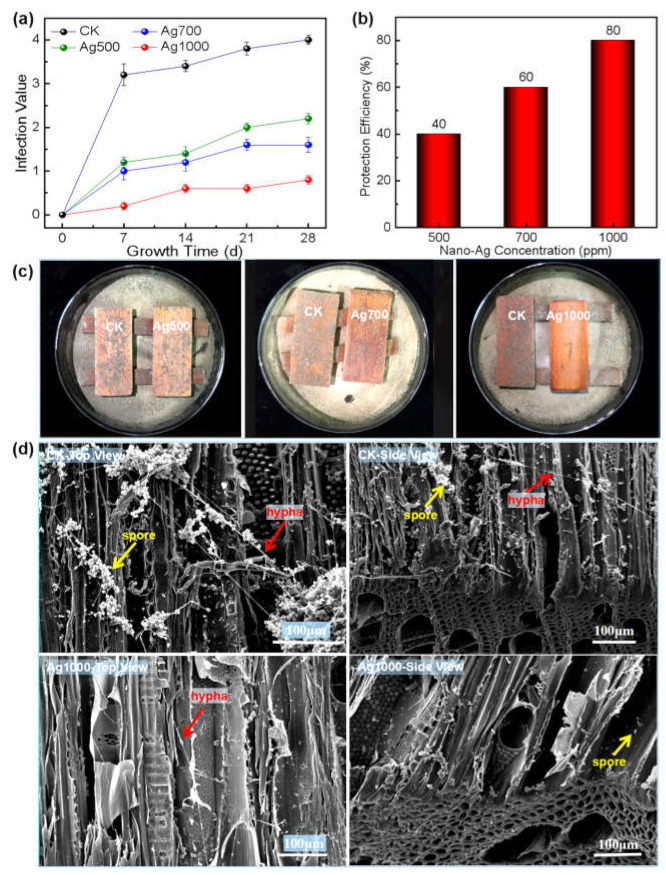
Growth inhibition of nano-silver against *Trichoderma viride* on wood surface: (**a**) Variation of infection values of *Trichoderma viride* on wood surface with time under different nano-silver concentrations (correspond to retentions, shown in Figure 2); (**b**) Protection efficiency of nano-silver against *Trichoderma viride* on wood surface under different nano-silver concentrations; (**c**) Digital photos of *Trichoderma viride* on wood surface after 28-day growth under different nano-silver concentrations (in the same Petri dish: left—CK; right—nano-silver treated sample); (**d**) SEM morphologies of the surfaces of CK wood and the wood treated by nano-silver with 1000 ppm concentration after *Trichoderma viride* infection.

Similar to the growth inhibition of the *Aspergillus niger* on wood surface, the growths of both *Penicillium citrinum* and *Trichoderma viride* on wood surface were also inhibited by the nano-silver; and the higher the concentrations/retentions of the nano-silver, the smaller the infection values of the two molds on wood surface (Figure 4a and Figure 5a), and thus the higher the protection efficiencies of the nano-silver against the mold fungi (Figure 4b and Figure 5b).

When the nano-silver concentration reached 1000 ppm, the protection efficiency of the wood surface against *Penicillium citrinum* and *Trichoderma viride* attained 75 and 80%, respectively, achieving or even exceeding the national standard value (75%, GB/T 18261-2013 [29]). Thus, 1000 ppm was the toxic threshold concentration of nano-silver against each *Penicillium citrinum* or *Trichoderma viride* infecting wood surface. Under this concentration, the mold fungi were almost inhibited with absent infection trace on the surface (Figure 4c and Figure 5c). Correspondingly, the SEM morphologies showed that compared with the CK wood, where abundant spores and hyphae of both the fungi appeared on the cell walls, there were few spores and hyphae present on the cell walls of nano-silver treated wood, suggesting that the nano-metal effectively inhibited the reproduction of both fungi on wood surface (Figure 4d and Figure 5d).

## 4. Discussion

### 4.1. The Leachability of Nano-Ag on Wood Surfaces

The antifungal element of the nano-metallic material existed at the nanoscale, with unique features of large specific surface area and abundant surface charges, possessing strong affinity with the polar components of wood, which could not be easily washed away by water flow, and thus exhibited higher leaching resistance than the correspondingly water-soluble ionic element [26]. Previous studies reported that the leaching rate of antifungal nanomaterials on wood surface was closely related to their physical and chemical environments, such as particle size, particle species, solution concentration, stabilizer type, wood species, water flow rate, water volume, pH value of leachate, light irradiating area, and ambient temperature [26,31,32]. In general, the larger the particle size, the more difficult it is for it to traverse within wood components, and the lower the leaching rate. It was reported that the leaching rate of nano-silver with, a diameter of 10 nm, reaches 12–16%, which was higher than that of the copper nanoparticle with a diameter of 50 nm, but was still much lower than the 24–57% of the ionic copper [26,31]. The leaching rate of nano-silver in this study was only 4.75% at 1000 ppm, which was comparable to the reported leaching rate (~4%) of nano-silver, but was lower than those of the above nano-copper and copper ions [31]. The information indicated that the nanoparticles should be less susceptible to water leaching, suggesting a higher leaching resistance than their ionic state.

The literature shows that the leachability of nanomaterials increases with their concentration or retention, due to the decreased active sites on wood surface [26,31]. Normally, wood cell wall components possess limited active sites and surface areas, and thus can only fix appropriate amounts of nanomaterials; with the increase in the surface retention, nanomaterials would aggregate into agglomerates with larger sizes, which accordingly weakens their adhesion onto wood surface [26,33]. In this study, SEM observation showed that in addition to the mono-dispersion on cell wall surface, nano-Ag particles also partially aggregated into agglomerates with particle size larger than 50 nm, which could weaken the interaction force between particles and wood components due to the reduction of specific surface area, and thus could be susceptible to leaching. The higher the concentration, the more the retention of nanomaterials on wood surface, and the greater the accessibility to water. With the immersion time extension, the loss amounts of nanomaterials would accordingly increase, which is reflected as the continuous increase in leaching rate. The experimental results showed that the leachability of the nano-Ag particles on wood surface increased with the increase in concentration (Figure 2), which was in accordance with the results reported in literature [26,31,34].

### 4.2. The Anti-Mold Effect of the Nano-Ag

**Figure 6:**
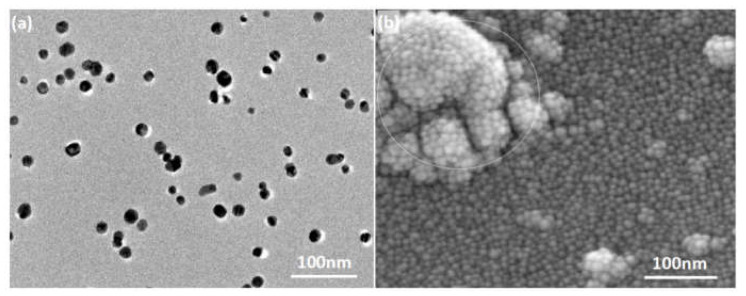
Morphologies of the nano-Ag agent: (**a**) TEM morphology of the nano-Ag (10 nm~20 nm) from Ag nanoparticles dispersion; (**b**) SEM morphology of the nano-Ag distributed on the surface of wood cell wall (1000 ppm).

The employed silver particle presented in a spherical form with a diameter ranging from 10 nm to 20 nm (Figure 6a), which corresponded to a huge specific surface area and thus guaranteed its nanoscale effect. Previous studies reported that in addition to the nanoscale effect contributing to anti-fungal activity, nanometallic particles mainly produced and released metal ions through morphological transformation to achieve their biocidal effectiveness; additionally, acidic environmental conditions could promote the transformation into ions for release, which accordingly increased the biocidal efficacy [26,31,35,36].

Therefore, after the nano-Ag being loaded onto wood surface, the weak acidic environment (pH = 4~6.5) of natural wood could theoretically promote the anti-fungal activity of the nano-Ag. SEM observation showed that the wood cell wall was fully covered by spherical nano-Ag particles with diameters ranging from 10 nm to 20 nm, positioning the nanoparticles so they were well distributed on wood surface in a mono-dispersed state, which would be beneficial to the nanoscale effect, and in return positively contribute to the inhibition efficacy of nano-Ag against the mold fungi. Accordingly, little infection of mold was observed on wood surfaces treated by the nano-Ag, as shown in Figure 3b, Figure 4c and Figure 5c.

It was reported that nano-silver destroyed fungal cell structure and inhibited mycelial growth as well as spore germination by making contact with the fungal cell surface via its affinity with the S and P elements [26,37,38,39]; and it could effectively inhibit fungal growth even at low concentrations, which was in agreement with the phenomenon of fewer spores and mycelium presented on the nano-silver treated wood surfaces, as shown in Figure 3d, Figure 4d, and Figure 5d. The nano-Ag strongly interacting with wood components guaranteed the uniform dispersion of nano-Ag particles and hereby resulted in a large specific surface area, which promoted the affinity of the nanomaterials with the fungi.

With the increase in nano-Ag concentration, the nanoparticle retention on wood surface positively increased, which was attributed to the free-flowing channels within wood and its huge specific surface areas, especially for poplar wood. However, Figure 2a showed a nonlinear relationship between nano-Ag retention and its concentration, which was stemmed from the heterogeneous and complex structure of wood. Such results were consistent with those reported in literature [26]. Additionally, an increase in nano-Ag retention accordingly contributed to the antifungal effect of the wood surface. The results of this study showed that there was almost a linear relationship between nano-silver concentration and the protective efficiency within the experimental range, i.e., the wood surface gained almost two-fold protective efficiency when the nano-Ag concentration increased from 500 ppm to 1000 ppm (Figure 3c, Figure 4b, and Figure 5b), and reached the required value of protective efficiency (75%) according to the national standard (GB/T 18261-2013 [29]).

In addition, in general, nanomaterials have small particle size, strong penetrating ability, and a strong interaction with wood components, which could easily confine the released antifungal ions into nanovoids within wood cell walls, and accordingly make it present high leaching resistance and biological durability [26].

## 5. Conclusions

This study explored using nano-silver to treat wood, and evaluated the relationships between the silver’s retention and the leachability or inhibitory effect, and thereby concluded as follows:

(1) The higher the nano-silver retention, the more powerful the anti-mold effect of the nanoparticle, and the larger the leachability with a slowly increasing tendency; when the nano-Ag retention on wood surface reached 0.324 g·m^−2^, its protection efficiency against the three molds exceeded 75%, and the leaching rate achieved merely 4.75%.

(2) The small-sized nano-silver was well distributed on wood surface in a mono-dispersed state, which promoted its contact probability with the molds and their interaction with the cell wall components, and thus ensured the anti-fungal effect and the leaching resistance of the nanoparticle.

(3) 1000 ppm was the toxic threshold concentration of the nano-silver (10 nm~20 nm) against all the three molds growth on the wood surface.

## Figures and Tables

**Table 1:** Classification of the infection value.

Infection Value	Infection Area
0	Without hypha on surface
1	Infection area < 1/4
2	Infection area 1/4~1/2
3	Infection area 1/2~3/4
4	Infection area > 3/4

## Data Availability

The data presented in this study are available on request from the corresponding author.
